# Comparison of Single- and Mixed-Sized Gold Nanoparticles on Lateral Flow Assay for Albumin Detection

**DOI:** 10.3390/bios11070209

**Published:** 2021-06-26

**Authors:** Sasima Chotithammakul, Michael B. Cortie, Dakrong Pissuwan

**Affiliations:** 1 Materials Science and Engineering Program, Faculty of Science, Mahidol University, Bangkok 10400, Thailand; sasima.cho19@gmail.com; 2 Nanobiotechnology and Nanobiomaterials Research Laboratory, School of Materials Science and Innovation, Faculty of Science, Mahidol University, Bangkok 10400, Thailand; 3 School of Mathematical and Physical Sciences, University of Technology Sydney, Broadway, NSW 2007, Australia; michael.cortie@uts.edu.au

**Keywords:** gold nanoparticles, single size, mixed sizes, lateral flow assay, albumin

## Abstract

The sensitivity and reproducibility of the lateral flow assay can be influenced by multiple factors, such as the size of gold nanoparticles (GNPs) employed. Here, we evaluated the analytical performance of single-sized and mixed-sized GNPs using a simple lateral flow assay (LFA) platform. This platform was used as a model assay to diagnose albumin levels and demonstrate the analytical performance of single-sized and mixed-sized GNPs in LFA tests. Two sizes of GNPs@anti-bovine serum albumin (BSA) conjugate proteins were mixed at different ratios. The unique optical properties of the GNPs induced a distinguishing color-shedding effect on the single- and mixed-sized GNPs@anti-BSA conjugates interacting with the target analyte BSA spotted on the test line. The use of mixed-sized GNPs@anti-BSA conjugates enhanced signal relative to the 20 nm GNPs, and provided superior stability compared with solely employing the large GNPs (50 nm). The proposed platform in this study could provide an efficient BSA detection mechanism that can be utilized as a model biomarker for confronting chronic kidney disease.

## 1. Introduction

Lateral flow immunoassay (LFA) is a simple, portable, and efficient platform for point-of-care testing. It can be used to diagnose various analytes, such as pathogens, antibiotics, and clinical target molecules [1,2,3]. Although LFAs provide many benefits, a sensitivity improvement of this technique is still required, therefore promoting the development of the LFA platform proposed in this study. Thus far, gold nanoparticles (GNPs) have been extensively applied for colorimetry purposes in LFAs because they exhibit a strong absorption of light in the green to red parts of the spectrum. The peak absorption wavelength depends upon particle diameter, shape, dielectric environment and state of aggregation, and can be red-shifted from 520 nm into the near-infrared. Increase in particle diameter exerts a strong non-linear increase on overall extinction. These topics have been extensively studied in the past, see ref. [4] for further background information. Since the color formation mechanism stems from light extinction induced by GNPs, the brightness of the detected color in LFAs depends on the amount of GNPs adsorbed on the membrane, thus indicating that the optical response of GNPs has a strong impact on detection sensitivity. The minimum number of particles required for achieving optical detection with naked eyes, and/or optical measuring devices, was also investigated by Khlebtsov et al. [5]. Accordingly, numerous studies have endeavored to optimize the size of GNPs to enhance the detection sensitivity of the LFA technique [6,7,8]. It was reported that different sizes of GNPs had different impacts on conjugation efficiency [9,10], and that when different sizes of GNPs are conjugated with the same molecules, the particles of larger size usually exert a high steric hindrance that can reduce an interaction between conjugated molecules and GNPs surface [9]. There have also been reports on the use of dual-size GNPs to increase the detection efficiency of GNP conjugate-based LFA techniques. As per the reports of Choi et al. [11], two sizes of GNPs were employed for conjugation with different target molecules—10 nm GNPs were conjugated with antibody molecules against analytes in the test sample, while 40 nm GNPs were conjugated with different molecules that could bind with the 10 nm antibody-conjugated GNPs mentioned above. The two types of conjugates were positioned in different areas, revealing that a dual system using two sizes of GNPs could induce a 100-fold enhancement in sensitivity, compared with the original LFAs. Another example of using a dual GNP system in LFA testing was reported by Chen et al. [12], where two sensing probes prepared from different-sized GNPs were used. The first probe comprised 28 nm GNPs conjugated with anti-*E. coli* (O157:H7) monoclonal antibodies, while the second probe comprised 45 nm GNPs conjugated with goat anti-mouse antibodies. Both probes reacted with the sample containing *E. coli* (O157:H7), resulting in the formation of an efficient complex that augmented the detection performance on the LFAs test line. These examples indicate that the use of different-sized GNPs in LFAs could advance their detection sensitivity.

**Figure 1:**
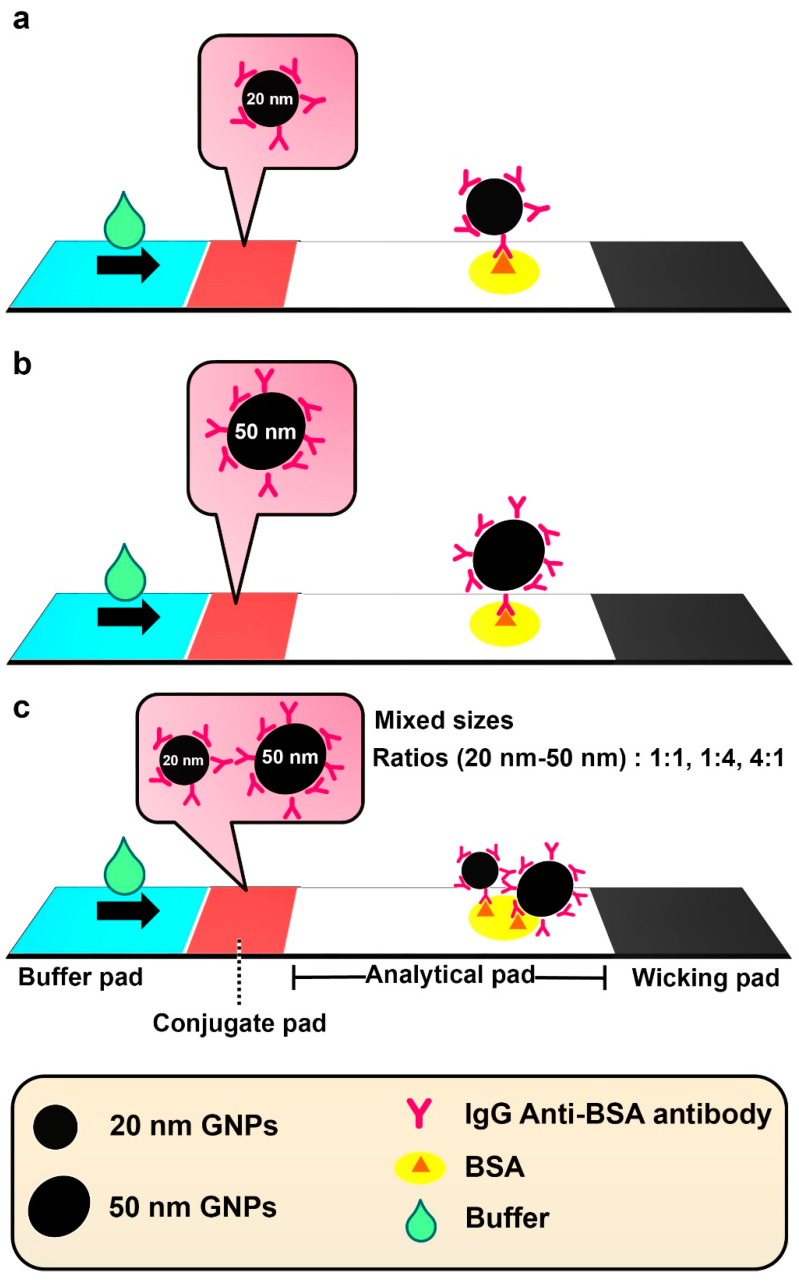
The schematic illustration of our proposed facile LFA strategy for BSA detection by employing GNPs conjugated with anti-BSA antibodies. Three GNP models were used: (**a**) single-sized (20 nm); (**b**) single-sized (50 nm); and (**c**) mixed-sized (20 nm and 50 nm) GNPs.

To the best of our knowledge, no reports exist so far on the application of mixed-sized GNPs, employing single conjugation with target molecules, as a detection probe in LFAs. Therefore, the idea of comparing and analytically evaluating the performance of traditional single-sized and fabricated mixed-sized single-ligand-conjugated GNPs for target molecule detection in LFAs was intriguing. Furthermore, the technique proposed here is more facile than conventional LFAs, requiring only the buffer to be applied on the sample pad, thereby enabling the direct detection of the analyte on the test line. Figure 1 schematically illustrates the function of the designed mixed-sized GNP LFA for BSA detection. Because serum albumin (SA) is a major circulatory protein present in the intravascular and interstitial spaces between cells, it was selected as the ideal target model to analytically evaluate the detection performance of our proposed strategy. Furthermore, SA can be used as a biomarker for the early detection of chronic kidney disease because it has been reported that a concentration of albumin in urea at ≥3.0 g dL^−1^ is closely associated with chronic kidney disease [13,14].

## 2. Material and Methods

### 2.1. Materials

Hydrogen tetrachloroaurate (III) trihydrate (HAuCl_4_·3H_2_O, 99.9%), trisodium citrate (Na_3_C_6_H_5_O_7_·2H_2_O), and anti-BSA antibodies produced in rabbits were purchased from Sigma-Aldrich (St. Louis, MO, USA). BSA was purchased from HIMEDIA (Mumbai, India). Phosphate buffer saline (PBS) without Ca^2^^+^ and Mg^2^^+^ was made by NacalaiTesque (Kyoto, Japan). Borate buffer (pH 7.4), boric acid (H_3_BO_3_), polyoxyethylene (20) sorbitan monolaurate, and sodium azide were purchased from OmniPur (Darmstadt, Germany). Trizma^®^ HCl·C_4_H_11_NO_3_; 99% and sucrose were purchased from MERK (Darmstadt, Germany). Millipore CO48 and backing cards were purchased from MERK (Darmstadt, Germany). STANDARD 14 and AE-99 were purchased from GE Healthcare (Buckinghamshire, UK). Supporter sheets and AE-99 were kindly supported by Bang Trading 1992 Co., Ltd. (Bangkok, Thailand).

### 2.2. Synthesis of GNPs

20 nm and 50 nm GNPs were synthesized using the Turkevich method [15]. Different concentrations of trisodium citrate at 77.6 mM and 19.4 mM were used for the synthesis of 20 nm and 50 nm GNPs, respectively. To synthesize the 20 nm GNPs, 5 mL of HAuCl_4_ solution (2 g L^−1^) was diluted in 45 mL of Milli-Q water. Subsequently, the prepared HAuCl_4_ was stirred while being heated until the temperature reached 95 °C. Following this, trisodium citrate (5 mL, 77.6 mM) was immediately added to the HAuCl_4_ solution and vigorous stirring was implemented until a ruby red color appeared. The solution was stirred under heat for 30 min, after which the heat source was removed. Furthermore, the solution was continuously stirred for another 30 min. Finally, the 20 nm GNPs were ready for use in the subsequent experiments. The preparation of the 50 nm GNPs was performed using a similar approach, with 2.5 mL of HAuCl_4_ solution (2 g L^−1^) added to 47.5 mL of Milli-Q water; different concentrations of trisodium citrate were used than those mentioned above. The prepared 20 nm and 50 nm GNPs were stored at 4 °C.

### 2.3. Characterization of the Synthesized GNPs (20 nm & 50 nm)

The characterization of the GNPs was performed using a UV-vis spectrophotometer (UV-2550, Shimadzu, Japan) to measure their plasmon resonance peaks. Transmission electron microscopy (TEM) was used to observe the morphology of both the GNP groups and measure their average sizes. The zeta potential values and the polydispersity indices (PDIs) were measured using a dynamic light scattering particle size analyzer. The PDIs, zeta potential values, and sizes were reported as mean ± standard deviation of the individual measurements.

### 2.4. Conjugation of GNPs with Anti-BSA Antibodies

25 μL aliquots of polyclonal anti-BSA antibodies produced in rabbits (anti-BSA antibody; 0.05 mg mL^−1^ in 1× PBS, pH 7.4) were directly mixed with 100 μL of the synthesized GNPs. The optical density (O.D.) was adjusted to 1.0 (at 521 nm for the 20 nm GNPs or at 530 nm for the 50 nm GNPs). To enhance the adsorption of anti-BSA antibodies to the surface of GNPs, the pH was adjusted at 8–9 during the binding reaction between the GNPs and anti-BSA antibody molecules. At this pH, the binding reaction was continued at room temperature for 15 min after mixing. Thereafter, the solution was centrifuged at 10,000 rpm (~9400× *g*) at 15 °C for 20 min (20 nm GNPs) or 10 min (50 nm GNPs). Following centrifugation, the supernatant was removed and the pellet was re-dispersed with 0.1× PBS buffer, pH 7.4, and stored at 4 °C for further use. It is worth noting that the small residual of unbound anti-BSA antibody can be blocked by the buffer used for conjugate pad preparation and the running buffer. Therefore, unbound antibodies that could impact proper interaction between the conjugate and analyte were eliminated. To confirm the conjugation, the plasmon resonance peak was measured using a NanoDropTM2000/c spectrophotometer.

### 2.5. Preparation of LFA Strip

The preparation of the LFA strip, as a single-step process, is illustrated in Figure 1. The strip comprised a buffer pad (Millipore CO-48 membrane; 0.5 cm × 1.7 cm), conjugate pad (Standard 14; 0.5 cm × 0.7 cm), analytical pad (a nitrocellulose AE-99, 0.5 cm × 2.5 cm), and wicking pad (Millipore CO-48 membrane; 0.5 cm × 1.7 cm). To prepare the conjugate pad, the solution of 20GNPs@anti-BSA, 50GNPs@anti-BSA or the mixed sizes of GNPs after conjugation with anti-BSA molecules at the ratios of 1:1, 1:4, and 4:1 (20GNPs@anti-BSA:50GNPs@anti-BSA) was diluted with 0.1× PBS buffer (pH 7.4) to obtain an O.D. of each plasmon resonance peak at 0.8. The conjugate pad was immersed into a solution of each conjugate before drying overnight at room temperature. After drying, the conjugate pad was soaked in 0.1 M Tris-HCl buffer, pH 8.3 (containing 10% sucrose, 0.25% sorbitan monolaurate), and was dried overnight at room temperature. The analytical pad was prepared on a support (vinyl card; 0.5 cm × 6 cm). BSA solution (1 μL) at different concentrations was blotted on the analytic pad, and the pad was dried overnight at room temperature. All prepared membranes were adhered on a backing card with an overlap area of each membrane at approximately 3 mm.

### 2.6. Sensitivity Determination and Specificity of the Prepared LFA

A buffer solution (0.1 M Tris-HCl buffer containing 1% Triton x-100, pH 8.3) was dropped onto the buffer pad to initiate the flow of the GNP probe to interact with the target BSA protein spotted on the analytical pad. Subsequently, the occurrence of signal amplification was detected on the analytical pad.

### 2.7. Data Analysis

After the reaction on the LFA was completed and the products were dried, a photograph was taken of the sensor using a Vivo 1935 mobile phone camera and fluorescent lighting. The color intensity on the analytical pad was measured on the photograph using Image J software. The same size of the rectangular area was set for measuring the color intensity on the membrane. The image was processed using RGB analysis with the background color of a blank membrane subtracted as a baseline. The line intensity profile and the integration of the area under curve were analyzed using the GraphPad Prism program. The ratios of the test line (TL) intensity vs. control line (CL) intensity of each strip were calculated and averaged. The measurements were repeated under all conditions in the analysis [16,17].

## 3. Results and Discussion

### 3.1. Characterization of GNPs

**Figure 2:**
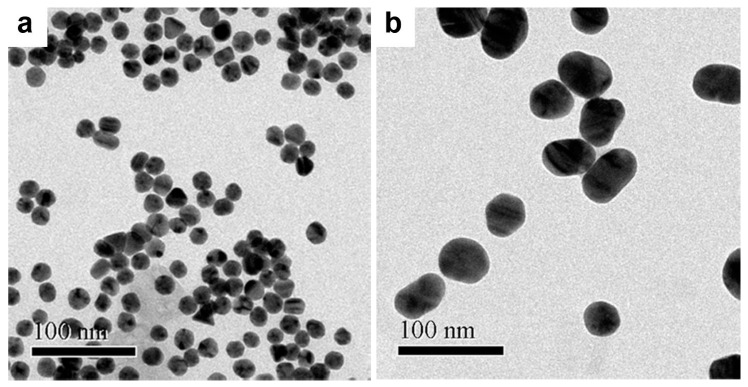
TEM images of (**a**) 20 nm and (**b**) 50 nm GNPs.

**Figure 3:**
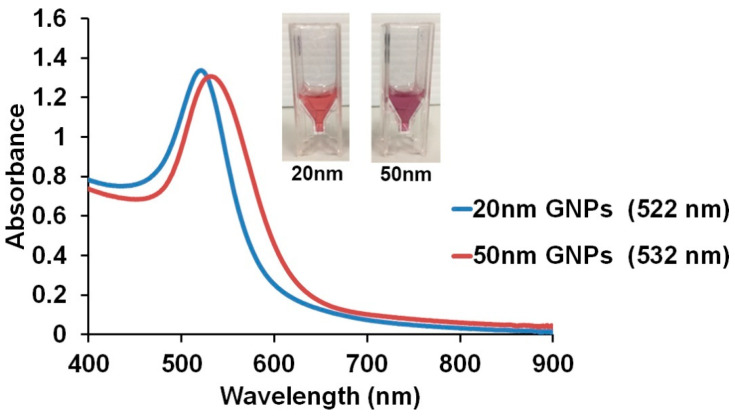
Light absorption spectra of the 20 nm and 50 nm GNPs.

The GNPs (20 and 50 nm) used in our work were synthesized using the well-known Turkevich method. The morphologies were analyzed by transmission electron microscopy (TEM) (Figure 2a,b). The 20 nm GNPs were uniform in size, the 50 nm GNPs somewhat less so. The 20 nm GNPs demonstrated a spherical shape (with an average size of ~19.6 ± 0.1 nm), while the 50 nm GNPs showed a slightly ellipsoidal shape (with the length of ~49.2 ± 0.9 nm and the width of 38.7 ± 0.6 nm). The light absorption spectra of the 20 nm and 50 nm GNPs are shown in Figure 3. The maximum light absorption of the 20 nm GNPs was at ~522 nm, while for the 50 nm GNPs it was at ~532 nm. The ellipsoidal shape of the 50 nm GNPs will have the effect that the plasmon resonance is split into a transverse and a longitudinal component, with peaks at slightly different wavelengths. The PDIs of the 20 nm GNPs and 50 nm GNPs were 0.29 ± 0.02 and 0.51 ± 0.01 respectively. The PDI can be used to predict the uniformity of the particles contained in the solution. It has been reported that PDIs above 0.7 indicate a polydisperse sample [18,19]. The zeta potentials of the 20 nm and 50 nm GNPs were −38.2 ± 2.8 and −41.2 ± 0.8 mV, respectively. The zeta potential distribution graph is shown in Supplementary Materials, Figure S1. The negative charge on the surface of both sizes GNPs were from citrate molecules on the surface of GNPs.

### 3.2. Bioconjugation of GNPs

**Figure 4:**
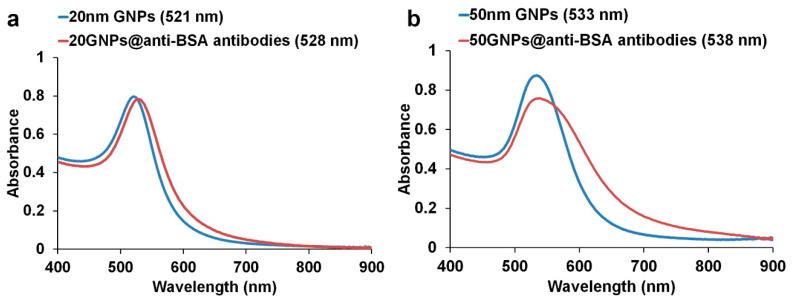
Comparison of the light absorption spectra of (**a**) 20 nm GNPs and (**b**) 50 nm GNPs before and after conjugation with anti-BSA antibodies.

Both sizes of GNPs were separately conjugated with anti-BSA antibody molecules through an electrostatic interaction, in a similar approach as the one used in our previous report [20]. This electrostatic interaction is formed between positively charged amino acids including N-terminal presenting in antibodies and negatively charged citrate-coated GNPs [21]. Following the conjugation process, the surface plasmon resonance (SPR) of the GNPs was red-shifted (from ~521 to ~528 nm for the 20 nm GNPs, and from ~533 to 538 nm for the 50 nm GNPs) (Figure 4a,b). This change suggested an increase in the refractive index of the surrounding medium, which was in agreement with the attachment of the anti-BSA antibody molecules on the GNPs surface [20,22]. Unlike their 20 nm equivalents, the 50 nm anti-BSA-antibodies-conjugated GNPs exhibited a broad absorption peak with decreased intensity, a clear indication of their instability [23].

Red-shifting and broadening of GNP plasmon peaks is often the result of aggregation. This generates a second, red-shifted, absorption peak in the spectrum, initially in the vicinity of 600 to 650 nm [24]. Here we searched for this occurrence by deconvoluting each spectrum into one or more Gaussian peaks, each corresponding to an individual resonance peak, as well as a Gaussian interband contribution at about 460 nm and a background component (See Supplementary Materials, Figure S2 for details). The spectrum for the conjugated 20 nm GNPs could be satisfactorily explained by a strong resonance with a peak at ~530 nm, plus a small peak (10% the height of the first one) at ~610 nm (see Supplementary Materials Figure S2). Each peak had a FWHM of about 0.09 eV. The second peak is very likely due to the formation of a small proportion of dimers in the suspension. Deconvolution of the spectrum of the conjugated 50 nm GNPs required the second peak (at 596 nm) to be 44% as high as the first peak. The FWHM of the peaks was 0.12 eV in this case. Given the observed instability of the colloid, it is also reasonable to assign the second peak to dimer formation (i.e., the early stages of aggregation). Note that the second peak in the 50 nm GNPs developed after conjugation with the BSA so cannot be principally due to a longitudinal plasmon resonance.

**Figure 5:**
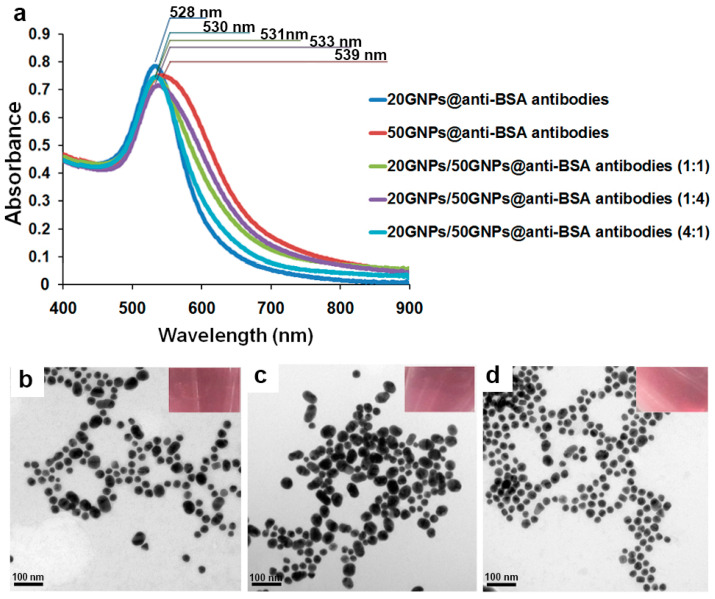
(**a**) Light absorption spectra of the 20GNPs@anti-BSA, 50GNPs@anti-BSA, and mixed-sized GNPs@anti-BSA conjugates. TEM images of 20GNPs@anti-BSA and 50GNPs@anti-BSA conjugates at different mixing ratios: (**b**) 1:1; (**c**) 1:4; and (**d**) 4:1.

The conjugation of GNPs with anti-BSA antibody molecules was referred to as 20GNPs@anti-BSA (for the 20 nm group) and 50GNPs@anti-BSA (for the 50 nm group). Initially, the optical density of both 20GNPs@anti-BSA and 50GNPs@anti-BSA was adjusted to 0.8, corresponding to each specific maximum absorption wavelength of conjugated particles. Subsequently, the GNPs were mixed at the ratios of 1:1, 1:4, and 4:1 (20GNPs@anti-BSA:50GNPs@anti-BSA). As expected, the maximum light absorption spectra of the mixed-sized GNPs@anti-BSA at different ratios changed from their original values (Figure 5a). These results confirmed that the mixture of two different sizes of GNPs was capable of affecting their plasmon resonances. The maximum light absorption spectra of the ratios were 531 (1:1 ratio), 533 (1:4 ratio), and 530 nm (4:1 ratio). It should be noted that the measured values of the surface plasmon resonances corresponding to the mixed-sized GNPs@anti-BSA at different mixing ratios ranged between the individual surface plasmon resonances of 20GNPs@anti-BSA (~528 nm) and 50GNPs@anti-BSA (~538 nm) in a relatively smooth progression, while contribution of the second plasmon peak also increased smoothly through the sequence (in Supplementary Materials, Figure S2). Furthermore, the mixed-sized GNP exhibited a narrower peak in the maximum light absorption spectra when compared with 50 nm GNPs alone (in Supplementary Materials, Figure S2). We also investigated the stability of different single- and mixed-sized GNPs@anti-BSA conjugates further (in Supplementary Materials, Figure S3). Here we define a ‘stable’ suspension as one that does not undergo any further aggregation or change over a seven day period. The results clearly show that 50GNPs@anti-BSA had a lower stability than that of mixed-sized GNPs@anti-BSA conjugates. If such instability were to also occur on the conjugate pad, then it could impact on sensitivity and accuracy of LFAs.

The TEM images of the mixed 20GNPs@anti-BSA and 50GNPs@anti-BSA conjugates at different mixing ratios, are shown in Figure 5b–d. As seen in Figure 5b, at a 1:1 ratio, the amounts of 20GNPs@anti-BSA and 50GNPs@anti-BSA conjugates appeared to be equal. Accordingly, when the ratio was changed to 1:4, more 50GNPs@anti-BSA conjugates were detected (Figure 5c). At a ratio of 4:1, the population of 20GNPs@anti-BSA conjugates was above that of the 50GNPs@anti-BSA conjugate population (Figure 5d). These observations confirmed that the mixture of conjugates was achieved at the desired ratios. To investigate whether the mixed-sized GNPs effectively enhanced the detection sensitivity performance, their signal amplification results were analyzed and discussed in the following section.

### 3.3. Evaluation of the Influence of Single- and Mixed-Sized GNPs@anti-BSA Conjugates on LFA Performance

**Figure 6:**
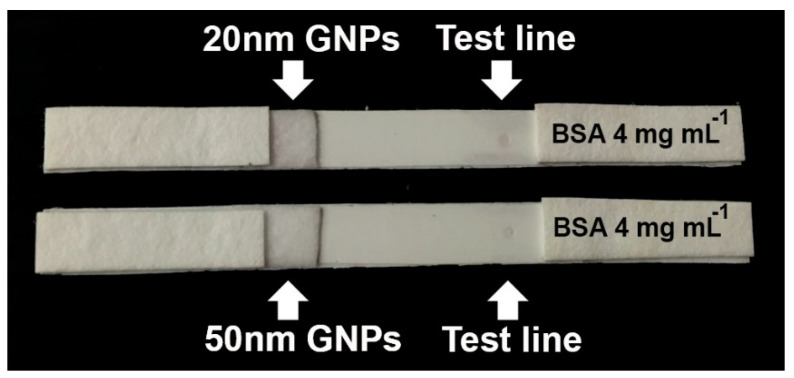
Use of non-conjugated GNPs (sizes of 20 and 50 nm) to detect spotted BSA molecules (concentration = 4 mg mL^−1^) on the test line.

It is well-known that nonspecific binding can lead to false positive, or even negative results. Therefore, we first investigated whether the unprocessed GNPs were solely capable of causing any nonspecific binding with the BSA molecules on the test line. For this task, non-conjugated GNPs (20 and 50 nm) were immobilized on the conjugate pad. Following this, the BSA solution (concentration = 4 mg mL^−1^) was spotted on the test line. As seen in Figure 6, there was no apparent color development on the test line, indicating that no interactions occurred between the GNPs and BSA molecules immobilized on the test line. As such, the GNPs were not involved in any nonspecific binding enhancement reactions.

**Figure 7:**
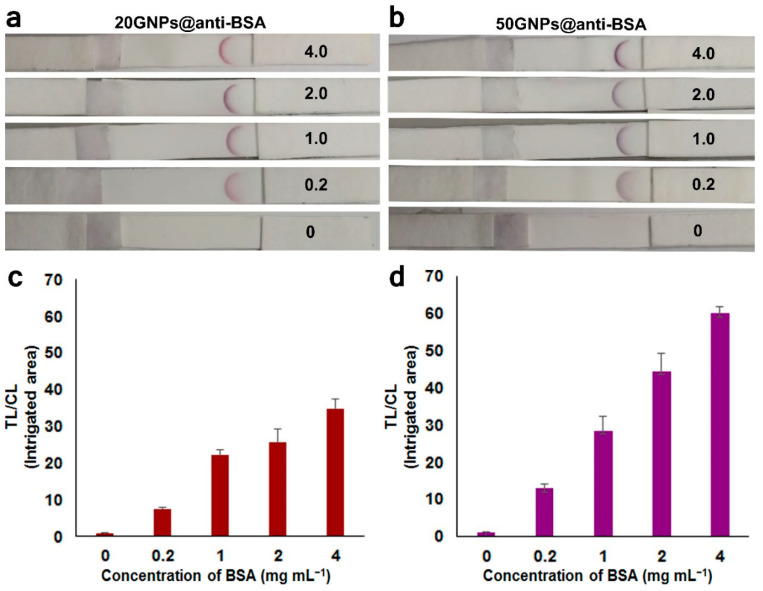
Detection process of BSA molecules with different concentrations (0, 0.2, 1, 2, and 4 mg mL^−1^) spotted on the test line of the LFA using 20GNPs@anti-BSA and 50GNPs@anti-BSA conjugates immobilized on the test pad as a detecting probe. (**a**,**b**) Visual detection of BSA molecules on the membrane using 20GNPs@anti-BSA or 50GNPs@anti-BSA conjugates, respectively. (**c**,**d**). Ratio of the test line intensity (TL) divided by the control line intensity (CL) showing detection signal intensity of BSA molecules using 20GNPs@anti-BSA and 50GNPs@anti-BSA conjugates, respectively. (n ≥ 3).

To evaluate the LFA sensitivity, 20GNPs@anti-BSA and 50GNPs@anti-BSA conjugates (both were adjusted to have an O.D. at 0.8) were separately immobilized on different pads for BSA detection. After the BSA solution (analyte; at different concentrations from 0.2 to 0.4 mg mL^−^^1^) was directly spotted on the test/analytical pad and the buffer solution was let to flow from the beginning of the pad, the conjugates could proceed to interact with the BSA molecules (target analytes) located on the test line (on which the BSA molecules could bind with the 20GNPs@anti-BSA and 50GNPs@anti-BSA conjugates flowing from the pad). The interacting conjugates with the BSA molecules were subsequently immobilized on the test line, to enable color development (Figure 7a,b). As expected, no color was developed on the test line segment without BSA molecules because the conjugates had no BSA molecules to bind with (Figure 7a,b). Among the BSA concentrations tested in this study (from 0.2 to 4.0 mg mL^−^^1^), the signal amplification mechanism procured a strong visual color at 4 mg mL^−^^1^; furthermore, the color development patterns at the test line were different according to the type of conjugate used. These results verified that the size of the GNPs affected the signal amplification of the LFA process. The impact of GNPs size on LFA detection performance was also reported by Kim et al. [7].

To qualitatively evaluate the collected data, the intensity profiles of the LFA strips were compared using the Color Profiler command of the Image J software. The peak areas at the test regions were calculated, along with the ratios of the test lines (TL) to the control lines (CL) of the intensity peaks. For both GNP groups (Figure 7c,d), the intensity of the TL to CL ratio increased with an increase in the BSA concentration. When comparing the individual contribution of each group on signal amplification for BSA detection, it was revealed that the signal from the 50GNPs@anti-BSA conjugates was stronger than that from the 20GNPs@anti-BSA conjugates. This was attributed to the larger GNPs size of the 50 nm GNPs, which endowed them with additional surface area to attach to the anti-BSA antibodies, thus increasing the reaction affinity to BSA species (as previously reported in [8]). It has been also reported that an even larger size of GNPs (e.g., 80 nm instead of 20) could further enhance the light absorption and scattering performance [25]. Furthermore, the slightly anisotropic optical properties of the 50 nm ellipsoidal GNPs might help increase signal amplification on LFAs [26]. Through the above investigation, the use of GNPs as a detection tool in LFAs can be systematically improved. The linear relationship between TL/CL intensity ratio and the concentration of BSA is shown in Supplementary Materials, Figure S4.

**Figure 8:**
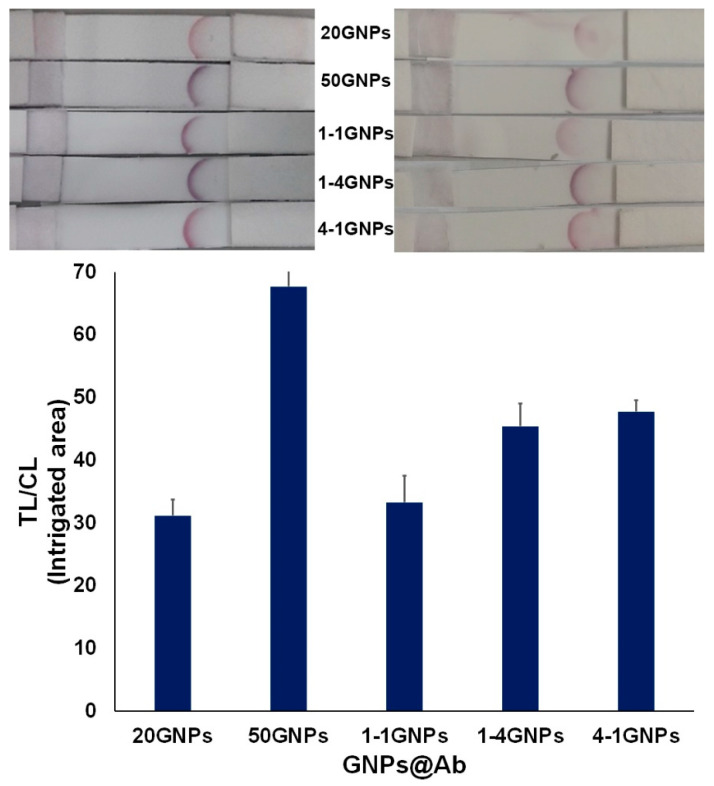
Ratio of the test line intensity (TL) divided by the control line intensity (CL) corresponding to each strip of particles immobilized on the conjugate pad for the detection of BSA molecules (4 mg mL^−1^). (n ≥ 3).

Through the employment of our proposed technique, BSA molecules with a concentration of 0.2 mg mL^−1^ could be effectively detected, with strong signal amplification for 4 mg mL^−1^ of BSA. The next question was how mixtures of the 20 and 50 nm GNPs would work, and whether they offered any additional advantage. Therefore, the approach of utilizing mixed-sized, instead of single-sized, GNPs@anti-BSA conjugates to detect BSA proteins in the 4 mg mL^−1^ solution was further investigated. For this purpose, mixtures of 20GNPs@anti-BSA and 50GNPs@anti-BSA conjugates, at ratios of 1:1, 1:4, and 4:1, were separately immobilized on the conjugate pad. The visual signals generated by mixing the two different-sized GNP groups at varying ratios produced two shades of colors on the test line, which could be attributed to the difference in SPR between the two groups (Figure 8). As previously mentioned, the use of 50 nm GNPs provided the optimal optical signal on the TL, which is in agreement with the general observation that the LFA sensitivity increases with the increase in the GNPs size [5]; however, the use of large-sized GNPs could potentially lead to instability which could have a detrimental impact on the efficiency of LFAs [12]. The negation of this phenomenon was the primary motive for our investigation on the combined utilization of different-sized GNPs in LFAs.

According to the TL/CL intensity ratio results (Figure 8), it was demonstrated that the GNP size ratio of the mixed conjugates also affected signal amplification. Specifically, when compared with solely using 20GNPs@anti-BSA conjugates, mixing the conjugates at the ratios of 1:4 and 4:1 (20GNPs@anti-BSA:50GNPs@anti-BSA) significantly contributed to the enhancement of the BSA molecules signal intensity, thus amplifying their detection probability. Our results demonstrated that the simultaneous use of two different-sized GNPs was viable.

The difference in signal detection performance between different-sized GNPs could be related to the number of binding molecules (anti-BSA antibodies in our research) attached on their surface. It was reported that an excessive concentration of a single binding molecule type over another could induce a decrease in the detection performance of GNP-based LFAs [27]. The varying detection signal intensity between different-sized (20 and 50 nm) GNP-conjugated particles, could be theoretically attributed to the different number of anti-BSA species that can be attached on their surface according to their size. It has been confirmed that the visual detection brightness depends on the number of adsorbed GNPs at the test spot (or line) of the LFAs [28]. There is also a possibility that mixed-sized GNPs conjugated with anti-BSA antibodies were assembled together at the test spot and helped improve localized surface plasmon transduction [29], resulting in enrichment of an optical label in LFAs. Therefore, this could constitute another reason why different visual signals were detected on the LFAs after employing different combinations of GNPs in terms of size and concentration ratio.

The strategy designed here was confirmed to benefit the detection procedure of BSA molecules, which are biomarkers of albuminuria. Although the highest TL to CL ratio value was measured for the 50GNPs@anti-BSA conjugates, they presented stability issues due to the larger GNP size (as previously discussed; Figures S2 and S3 in Supplementary Materials). Instability of conjugated GNPs, whether on the pad prior to use or when the buffer finally mobilizes the GNP during the test, could impact on the quality of the optical signal in LFAs. Therefore, it is helpful to minimize instability of the conjugated GNPs from the very beginning of the design process. The use of mixed-sized GNPs provides an approach for mitigating the complication of instability, which stems from conjugating large-sized GNPs with biomolecules.

## 4. Conclusions

GNPs possess distinct optical properties for biological detections [30,31,32]. The conjugation of single and mixed-sized GNPs with anti-BSA antibodies can effectively augment the detection performance of LFAs. This provides a promising strategy for the rapid and facile detection of BSA molecules, which constitute markers for early signs of chronic kidney disease. When comparing the overall performance between single-sized and mixed-sized GNPs used in our experiments, it was revealed that the mixed-sized GNPs (namely 20 and 50 nm) produced a stronger signal than that obtained using the 20 nm GNPs alone. In addition, the stability of the mixed-sized GNPs was superior to that obtained using 50 nm GNPs alone, as indicated by the former’s narrower resonance peak. Our proposed strategy combines the principles of colorimetric detection and the advantages of the simple LFA technique, with the purpose of screening the concentration of BSA (or other target analytes) in a tested biosystem sample. Moreover, it provides a straight-forward approach without using a multi-step process.

## Data Availability

Not applicable.

## Supplementary Materials

The following are available online at https://www.mdpi.com/article/10.3390/bios11070209/s1, Figure S1: The zeta potential distribution graphs of (**a**) 20 nm GNPs and (**b**) 50 nm GNPs. Figure S2: Optical spectra of single- and mixed-sized GNPs@anti-BSA conjugates after deconvolution. Figure S3: The relative optical density of single- and mixed- sized GNPs@anti-BSA conjugates. Figure S4: The linear relationship between TL/CL intensity ratio and the concentration of BSA of (a) 20GNPs@anti-BSA conjugates and (b) 50GNPs@anti-BSA conjugates.
