# Supplementary material for: Comparison of Single- and Mixed-Sized Gold Nanoparticles on Lateral Flow Assay for Albumin Detection
Source: Biosensors (Basel). 2021 Jun 26;11(7):209. doi: 10.3390/bios11070209 (PMC8301757; doi:10.3390/bios11070209)
Supplement: Supplementary file 1 [file biosensors-11-00209-s001.zip › biosensors-1225890-supplementary.pdf]

biosensors

Supplementary Material

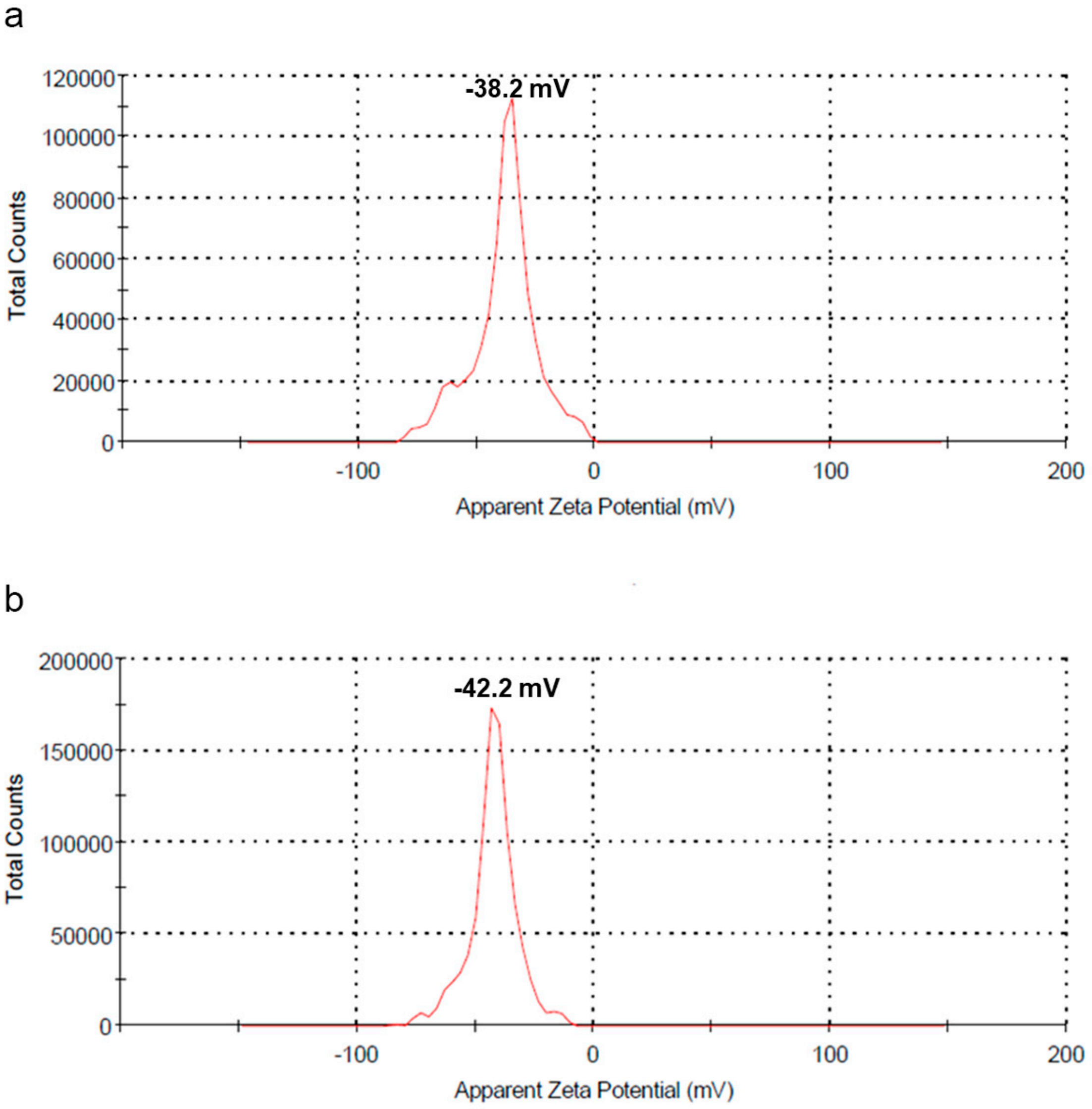

Figure S1. The zeta potential distribution graphs of (**a**) 20 nm GNPs and (**b**) 50 nm GNPs.

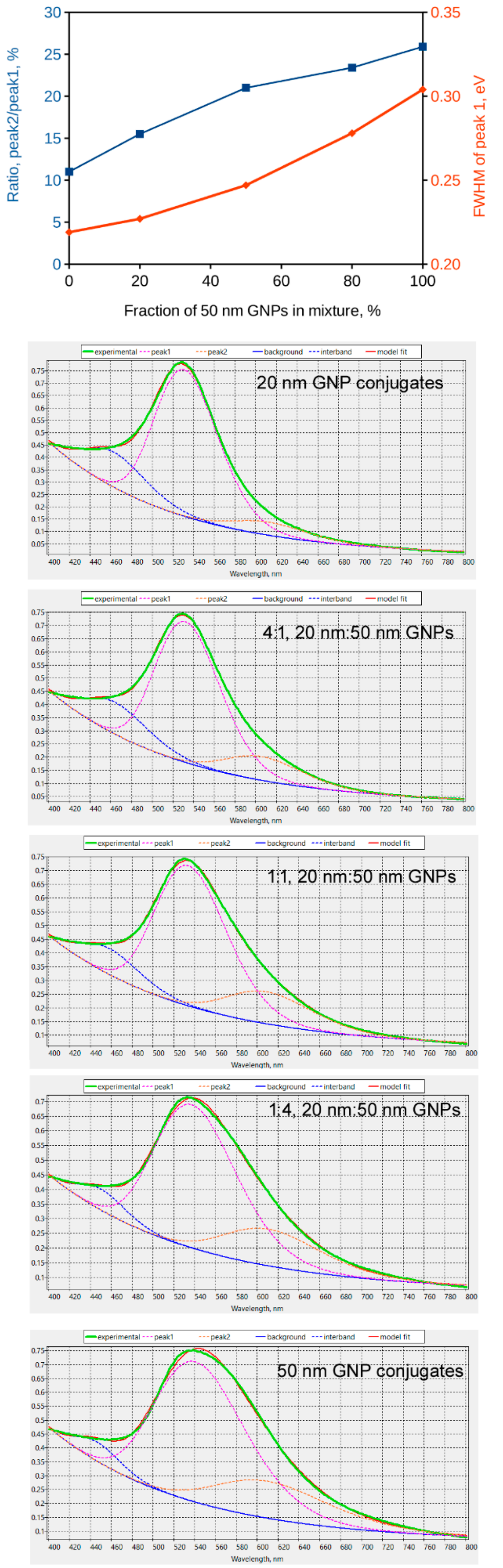

Figure S2. Optical spectra of single- and mixed- sized GNPs@anti-BSA conjugates after deconvolution.

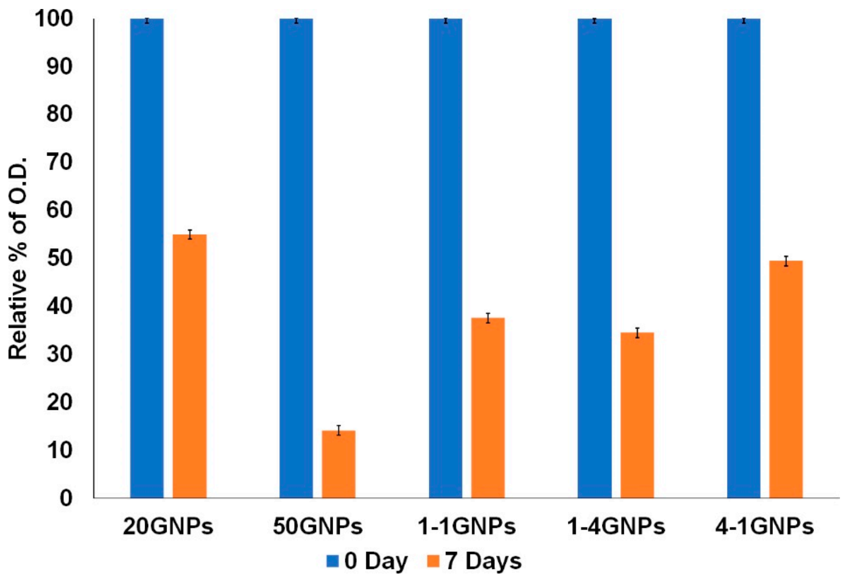

Figure S3. The relative optical density of single- and mixed- sized GNPs@anti-BSA conjugates.

After 7 days investigation of single- and mixed- sized GNPs@anti-BSA conjugates stability. It clearly shows that the optical density of 50GNPs@anti-BSA conjugates reduced from its original value. This reduction reveals a decrease in stability of 50GNPs@anti-BSA conjugates. The stabilities of mixed-sized GNPs@anti-BSA conjugates at the ratios of 1:1, 1:4, and 4:1 (20GNPs@anti-BSA:50GNPs@anti-BSA) were better than that of 50GNPs@anti-BSA conjugates.

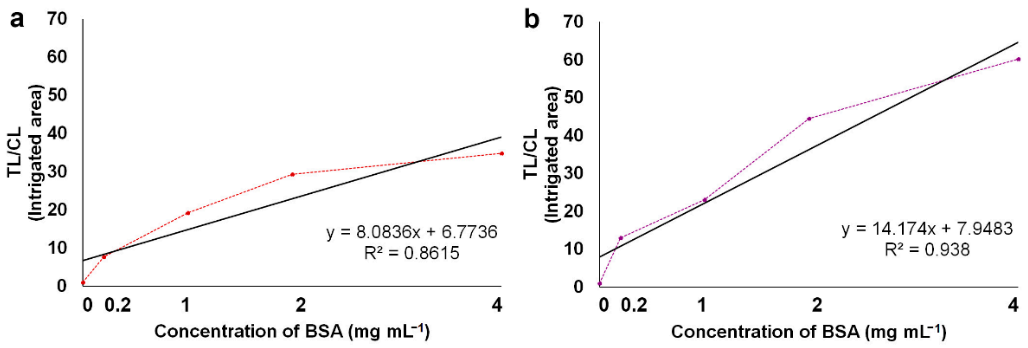

Figure S4. The linear relationship between TL/CL intensity ratio and the concentration of BSA of (a) 20GNPs@anti-BSA conjugates and (b) 50GNPs@anti-BSA conjugates.

As expected, the use of 50GNPs@anti-BSA conjugates provided a better linearity in the range from 0 to 4 mg mL^{-1} BSA than that of using 20GNPs@anti-BSA conjugates.
